# The FVB Background Does Not Dramatically Alter the Dystrophic Phenotype of Mdx Mice

**DOI:** 10.1371/currents.md.28266819ca0ec5fefcac767ea9a3461c

**Published:** 2015-02-10

**Authors:** Nalinda B. Wasala, Keqing Zhang, Lakmini P. Wasala, Chady H. Hakim, Dongsheng Duan

**Affiliations:** Department of Molecular Microbiology and Immunology, University of Missouri, Columbia, Missouri, USA; Department of Molecular Microbiology and Immunology, University of Missouri, Columbia, Missouri, USA; Department of Molecular Microbiology and Immunology, University of Missouri, Columbia, Missouri, USA; Department of Molecular Microbiology and Immunology, University of Missouri, Columbia, Missouri, USA; Department of Molecular Microbiology and Immunology, University of Missouri, Columbia, Missouri, USA

## Abstract

The mdx mouse is the most frequently used animal model for Duchenne muscular dystrophy (DMD), a fatal muscle disease caused by the loss of dystrophin. Mdx mice are naturally occurring dystrophin-null mice on the C57BL/10 (BL10) background. We crossed black mdx to the white FVB background and generated mdx/FVB mice. Compared to that of age- and sex-matched FVB mice, mdx/FVB mice showed characteristic limb muscle pathology similar to that of original mdx mice. Further, the forelimb grip strength and limb muscle (tibialis anterior and extensor digitorum longus) specific force of mdx/FVB mice were significantly lower than that of wild type FVB mice. Consistent with what has been reported in original mdx mice, mdx/FVB mice also showed increased susceptibility to eccentric contraction-induced force loss and elevated serum creatine kinase. Our results suggest that the FVB background does not dramatically alter the dystrophic phenotype of mdx mice.

## Introduction

Duchenne muscular dystrophy (DMD) is an X-linked lethal progressive muscle wasting disorder mainly affecting boys. It is caused by mutations in the dystrophin gene, one of the largest and most conserved genes in the genome (reviewed in 1 ). Numerous mouse models have been used to study dystrophin function and DMD pathogenesis (reviewed in 2 ^-^ 5). Among these, the mdx mouse is the most frequently used model (reviewed in 6). First described in 1984 by Bulfield and colleagues as a spontaneous myopathy model in C57/BL10 (BL10) mice, mdx mice carry a nonsense mutation in the exon 23 of the dystrophin gene 7 ^,^ 8.

Transgenesis is one of the most powerful technologies to investigate gene function in animal models (reviewed in 9 ^,^ 10). Studies conducted in transgenic mdx mice have laid the foundation for our current understanding of the structure-function relationship of dystrophin and DMD gene therapy (reviewed in 11). We recently began to use the transgenic approach to characterize the function of the dystrophin nNOS-binding domain and to explore cardiac unique features of the dystrophin gene 12,sup>-15. The FVB mouse has been the preferred inbred strain for the production of transgenic mice because of its robust reproductive performance, unusually large pronuclei of the fertilized oocytes (easy for microinjection) and excellent nurturing characteristics 16. To minimize the influence of the genetic background, in the past we have to backcross our FVB background transgenic mice for many generations to the BL10 background before use them in the study 12 ^-^ 14. With more transgenic lines being developed, it becomes an extremely labor-intensive and time-demanding task to backcross every FVB transgenic line to the mdx background. To solve this problem, we decided to generate FVB background mdx (mdx/FVB) mice. We backcrossed inbred FVB mice with the original BL10 background mdx mice for seven generations. The resulting mdx/FVB mice had white coat color. However, they showed the characteristic histological and physiological changes as the original mdx mice. Our results suggest that the mdx/FVB mouse may represent a useful model to study DMD.

## Materials and Methods

**Experimental Animals. **All animal experiments were approved by the institutional animal care and use committee and were in accordance with NIH guidelines. Parental FVB (FVB/NJ, Jackson stock number 001800) and mdx (C57BL/10ScSn-Dmd^mdx^/J, Jackson stock number 001801) mice were obtained from The Jackson Laboratory (Bar Harbor, ME). All mice were maintained in a specific-pathogen free animal care facility on a 12-hour light (25 lux): 12-hour dark cycle with access to food and water *ad libitum*. For histological and physiological studies, only male mice were used.

**Generation of mdx/FVB mice. **The mdx/FVB mouse was generated by seven generations of backcrossing. Briefly, female mdx mice were crossed with male FVB mice to obtain the F1 progeny. Heterozygous females were bred with FVB males to get F2 progeny. Heterozygous F2 females were identified by PCR according to our published protocol and crossed with male FVB mice to get F3 progeny 17. In subsequent rounds of breeding, the heterozygous females were used to cross with FVB males until a total of seven generations of backcrossing was finished. Dystrophin-deficient males and heterozygous females from the last round of backcrossing were interbred to generate experimental mdx/FVB mice.

**Morphological studies**. General histology was examined by hematoxylin and eosin (HE) staining. Central nucleation was quantified on 3 to 5 random 20x fields for each muscle. Fibrosis was examined by Masson trichrome staining as we described before 18. Dystrophin expression was evaluated by immunofluorescence staining using the dystrophin C-terminal domain specific Dys-2 antibody (1:100; clone Dy10/12B2, IgG2a; Novocastra, Newcastle, UK) 19 ^,^ 20. Slides were viewed at the identical exposure setting using a Nikon E800 fluorescence microscope. Photomicrographs were taken with a Qimage REtiga 1300 camera 18.

**Serum creatine kinase (CK) activity assay. **Fresh serum was collected by tail vein bleeding. The CK activity was determined using CK liqui-UV test kit from Stanbio Laboratory (Boerne, TX) according to the manufacturer’s guidelines.

**Fore limb grip strength measurement. **Fore limb grip strength was measured with a computerized grip strength meter (Columbus Instruments, Columbus, OH) as we described previously 21 ^,^ 22. The grip strength meter has a pulling bar attached to a force transducer and a digital display. Briefly, the mice were first checked for any sores in the limbs and toes prior to the experiment. Only mice without apparent skin injury were used in the study. The mice were first acclaimed to the apparatus for approximately 5 min. Mouse was then allowed to grab the pulling bar by holding it from the tip of the tail. The mouse was gently pulled away from the grip bar. When the mouse can no longer grasp the bar, the reading was recorded. Protocol was repeated five times with at least 30 sec rest between trials. The highest three values were averaged to obtain the absolute grip strength. Normalized grip strength was obtained by dividing the absolute grip strength with the body weight.

**EDL muscle function evaluation. **EDL muscle force was determined ex vivo according to our published protocol 21 ^,^ 23. Briefly, mice were anesthetized via intra-peritoneal injection of a cocktail containing 25 mg/ml ketamine, 2.5 mg/ml xylazine and 0.5 mg/ ml acepromazine at 2.5 µl/g body weight. The EDL muscle was gently dissected and mounted to an intact muscle test system (Aurora Scientific, Inc., Aurora, ON, Canada) containing oxygenated (95% O_2_ and 5% CO_2_ at 30ºC) Ringer’s buffer. After 10 min equilibration, the muscle length (L_m_) of the EDL muscle was measured with an electronic digital caliper (Fisher Scientific, Waltham, MA, USA). This length is defined as the optimal muscle length (L_0_). The maximum isometric tetanic force (Po) was measured at 150 Hz. The muscle cross-sectional area (CSA) was calculated according to the following equation, CSA = (muscle mass, in gram)/[(optimal fiber length (L_f_), in cm) × (muscle density, in g/cm3)]. A muscle density of 1.06 g/cm3 was used in calculation. The optimal fiber length is calculated as 0.44 x Lo. 0.44 represents the ratio of the fiber length to the Lo of the EDL muscle 21. Specific muscle force was determined by dividing the maximum isometric tetanic force with the muscle CSA. After tetanic force measurement, the muscle was rested for 10 min and then subjected to ten rounds of eccentric contraction injury according to our previously published protocol 21 ^,^ 23. Briefly, following a tetanic contraction EDL muscle was stretched 10%L_0 _at a rate 0.5L_0_/sec. The muscle was allowed to rest 2 min between each eccentric cycle. The percentage of force drop following each round of eccentric contraction was recorded. Data were processed using the Lab View-based DMC and DMA programs (Version 3.12, Aurora Scientific, Inc.).

**TA muscle function evaluation. **The TA muscle force was measured in situ according to our published protocol 21 ^,^ 23. Briefly, mice were anesthetized as described above. The TA muscle and the sciatic nerve were exposed. The mouse was transferred to a customer-designed thermo-controlled platform of the footplate apparatus. Sciatic nerve was stimulated using a custom-made 25G platinum electrode to elicit muscle contraction. Subsequently, twitch and tetanic forces and the eccentric contraction profile were measured with a 305C-LR dual-mode servomotor transducer (Aurora Scientific, Inc.). Data recording and analysis were identical to methods described for the EDL muscle. In TA muscle cross-sectional area calculation, the optimal fiber length was calculated as 0.60 x L_0_. 0.60 represents the ratio of the fiber length to the L_0 _of the TA muscle 21.

**Statistical analysis. **Data are presented as mean ± standard error of mean (s.e.m.). Statistical significance between FVB and mdx/FVB was determined by the Student t-test. Difference was considered statistically significant when *P *< 0.05.

## Results

**Adult mdx/FVB mice show dystrophic muscle pathology and elevated serum CK. **The coat color of mdx mice is black while that of FVB mice is white. After crossing mdx mice with FVB mice for seven generations, we obtained the expected white colored mdx/FVB mice (Figure 1A). To determine whether mdx/FVB mice had myopathy, we first examined histology in the TA muscle of 3 and 6-m-old mice (Figure 1B). Dystrophin expression was observed in the FVB muscle but not in the mdx/FVB muscle. On HE staining, we observed inflammation, degeneration/regeneration and necrosis in the mdx/FVB muscle (Figure 1B). The FVB muscle had the uniform myofiber size but in the mdx/FVB muscle, we noticed the presence of extremely large and small myofibers (Figure 1B). In wild type FVB mice, central nucleation was <1%. In mdx/FVB mice, central nucleation reached 66-71% (Table 1, N=11 mice/group, ~ 12,000 myofibers quantified per strain).

**The tibialis anterior (TA) muscle of the mdx/FVB mouse displays characteristic histological changes of muscular dystrophy.**
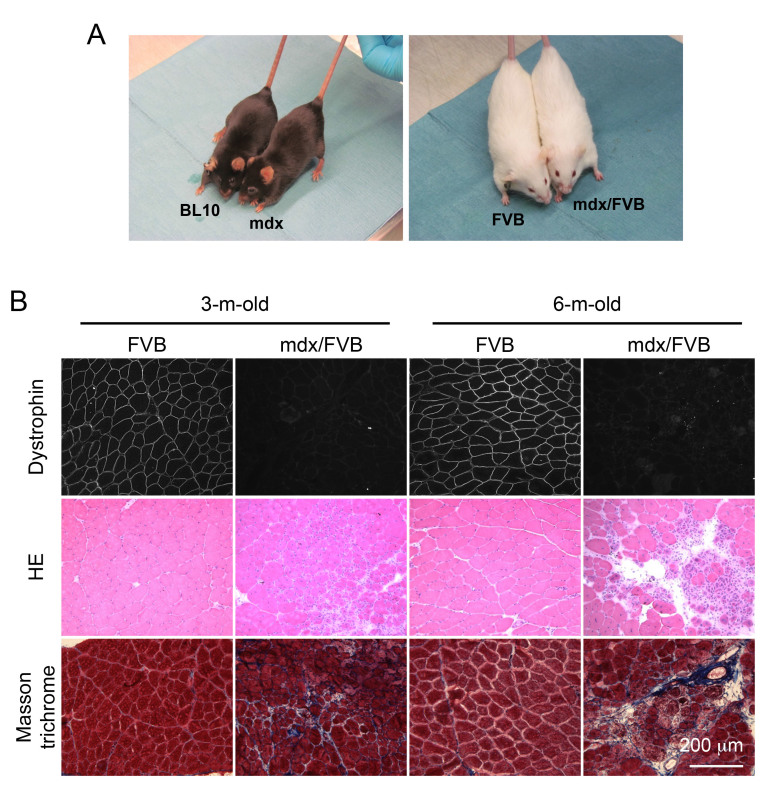
**A**, Representative photographs of experimental mice. Left panel, BL10 and mdx mice; Right panel, FVB and mdx/FVB mice. **B**, Representative photomicrographs of dystrophin immunofluorescence staining (top panel), HE staining (middle panel) and Masson trichrome staining (bottom panel) of the FVB and mdx/FVB TA muscles.

CK level elevation is a salient feature in mdx mice. Consistently, the CK level in mdx/FVB mice was also significantly higher than that of FVB mice (Figure 2A).

**Muscle function is significantly compromised in mdx/FVB mice. **Three methods were used to evaluate muscle function in 3 and 6-m-old FVB and mdx/FVB mice. Forelimb grip strength was quantified in awaken intact mice. Compared to that of FVB mice, body-weight normalized grip strength was reduced by ~ 50% in mdx/FVB mice (Figure 2B).

**Serum creatine kinase (CK) and forelimb grip in FVB and mdx/FVB mice.**
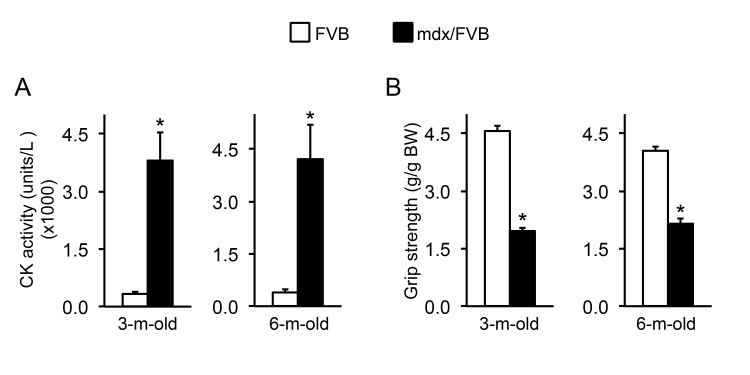
**A**, Quantification of the serum CK level. n=8 and 9 for 3-m-old and n=4 and 8 for 6-m-old FVB and mdx/FVB mice, respectively. **B**, Forelimb grip strength. n=7 and 8 for 3-m-old and n=4 and 6 for 6-m-old FVB and mdx/FVB mice, respectively. The absolute grip force is normalized to the body weight. Asterisk, significantly different from that of FVB mice.

The TA muscle force was analyzed in situ in anesthetized mice (Figure 3, Table 1). The specific twitch force was marginally reduced in mdx/FVB mice (p=0.05) (Figure 3A). However, the specific tetanic force was significantly decreased in mdx/FVB mice. It only reached ~ 80% of the normal (Figure 3B). Mdx/FVB mice were significantly more susceptible to eccentric contraction damage (Figure 3C). From 3 to 6 months, the eccentric contraction profile of FVB mice did not change much. Interestingly, compared to that of 3-m-old mdx/FVB mice, 6-m-old mdx/FVB mice showed a much sharper force drop during the first three rounds of eccentric contraction. The residual force was also lower in 6-m-old mdx/FVB (~21% of the starting force; this value was ~ 35% in 3-m-old mdx/FVB).

**In situ analysis of the contractile properties of the tibialis anterior (TA) muscle in FVB and mdx/FVB mice.**
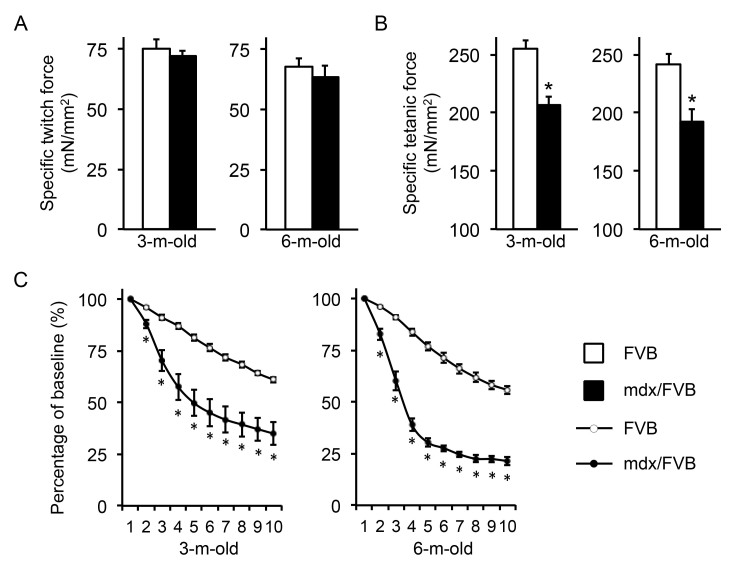
**A**, Specific twitch force. n=10 and 8 for 3-m-old and n=8 and 9 for 6-m-old FVB and mdx/FVB mice, respectively; p=0.51 and p=0.50 for 3 and 6-m-old comparisons respectively. **B**, Specific tetanic force. n=10 and 8 for 3-m-old and n=8 and 9 for 6-m-old FVB and mdx/FVB mice, respectively. **C**, Percentage of force drop during ten cycles of eccentric contractions. Absolute force generated during the first cycle is set as the baseline (100%) and the percentage of force drop following each cycle of eccentric contraction is calculated relative to the baseline. n=7-10 and 7-8 for 3-m-old and n=7-8 and 6-9 for 6-m-old FVB and mdx/FVB mice, respectively. Asterisk, significantly different from that of FVB mice.

The EDL muscle force was analyzed ex vivo (Figure 4, Table 1). The overall trend was similar to that of the TA muscle with mdx/FVB mice showing significantly more compromised contractility compared to that of normal mice. Interestingly, a statistically significant difference in the specific twitch force was found between FVB and mdx/FVB mice (Figure 4A). Further, the eccentric contraction profile of 3-m-old mdx/FVB mice was similar to that of 6-m-old mdx/FVB (Figure 4C).

**Table 1. Comparison of body weight, muscle weight and centronucleation in FVB and mdx/FVB mice:** Data shown as mean ± standard error of mean. a, significantly different from the 3-m-old FVB control group; b, significantly different from the 6-m-old FVB control group. Abbreviations: EDL, extensor digitorum longus; CSA, cross sectional area; TA, tibialis anterior; CN, central nucleation. Sample size: Body weight n=11-14 for FVB, n=11-19 for mdx/FVB; EDL weight/CSA n=14-24 for FVB, n=12-18 for mdx/FVB; TA weight/CSA n=8-10 for FVB, n=8-9 for mdx/FVB; CN analysis n= 10-11 for each strain.

	FVB		mdx/FVB	
	3-m-old	6-m-old	3-m-old	6-m-old
Body weight (g)	28.67±0.60	32.52±0.44	34.06±0.58^a^	39.19±1.20^b^
EDL weight (mg)	11.08±0.27	12.36±0.16	14.03±0.14^a^	13.41±0.19^b^
EDL CSA (mm2)	1.81±0.04	1.95±0.03	2.13±0.05^a^	2.07±0.04^b^
TA weight (mg)	52.92±1.45	65.38±1.04	62.24±3.01^a^	73.99±2.10^b^
TA CSA (mm2)	5.63±0.15	6.90±0.12	6.08±0.29	7.29±0.14^b^
Central nucleation (%)	0.28±0.15	0.19±0.03	65.80±1.44^a^	70.77±0.82^b^

**Ex vivo analysis of the contractile properties of the extensor digitorum longus (EDL) muscle in FVB and mdx/FVB mice.**
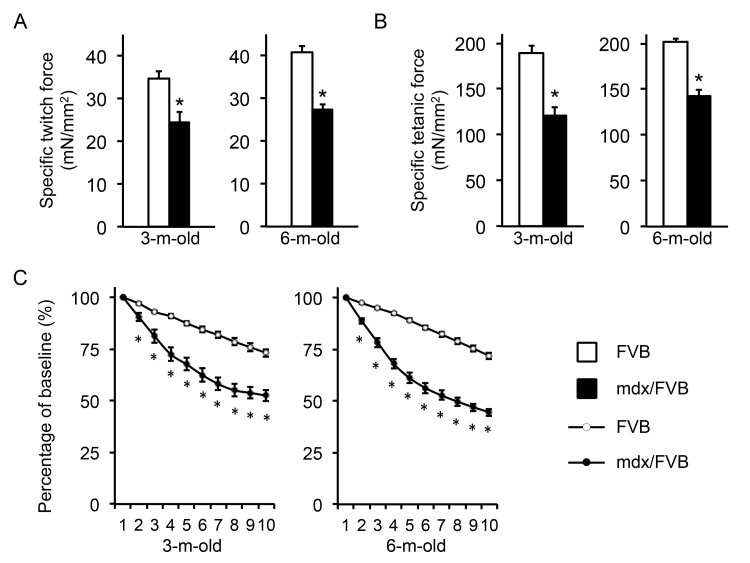
**A**, Specific twitch force. n=14 and 12 for 3-m-old and n=24 and 18 for 6-m-old FVB and mdx/FVB mice respectively. **B**, Specific tetanic force. n=14 and 12 for 3-m-old and n=24 and 18 for 6-m-old FVB and mdx/FVB mice respectively. **C**, Percentage of force drop during ten cycles of eccentric contractions. Absolute force generated during the first cycle is set as the baseline and (100%) and the percentage of force drop following each cycle of eccentric is calculated relative to the baseline. n=9-12 and 9-12 for 3-m-old and n=14-15 and 12-14 for 6-m-old FVB and mdx/FVB mice respectively. Asterisk, significantly different from FVB mice.

## Discussion

To meet the practical needs of our transgenic studies, we crossed the BL10-background mdx mice with FVB/NJ mice. Recent studies suggest that the so-called “wild type” inbred mice may actually carry various changes in their genome. For example, the commonly used A/J mice were recently show to display progressive muscular dystrophy due to a mutation in the dysferlin gene 24. The FVB strain was also found to carry mutations in several genes of the visual system 25. It is thus important to determine whether the FVB background alters the dystrophic phenotype of the original mdx mice. After seven generations of backcross, we obtained white mdx/FVB mice. These mice showed classic dystrophic changes including elevated serum CK, myofiber centronucleation, muscle inflammation and fibrosis, force reduction and enhanced sensitivity to eccentric contraction injury. In young adult mdx mice, the specific twitch and tetanic force for EDL muscle range from 26.6±1.2 to 29.0±1.5 and 129.6±10.5 to 138.5±5.6 mN/mm^2^, respectively. In young adult C57Bl/10 (BL10) mice, the specific twitch and tetanic force for EDL muscle range from 33.2±1.8 to 46.5±3.7 and 185.4±5.7 to 245.0±1.4 mN/mm^2^, respectively 12 ^,^ 26 ^-^ 28. Muscle force drops from 100% (baseline) to 53.5-29.4% (after 10cycles of eccentric contraction) in young mdx mice. Muscle force drops from 100% (baseline) to 73.1-68.0% (after 10 cycles of eccentric contraction) in BL10 mice 26 ^,^ 27. The values we observed in EDL muscle of mdx/FVB mice were comparable to these of mdx mice. The contractile properties of the TA muscle in mdx/FVB mice and FVB mice also fall within the range of those reported in mdx/mdx4cv mice and BL10/BL6 mice, respectively 29 ^-^ 31. Tables 2 and 3 show a comprehensive comparison of contractile properties of limb muscles in FVB vs BL10 and mdx/FVB vs mdx/BL10 mice. Elevated levels of serum CK and myofiber centronucleation are hallmarks of muscle diseases in mdx and mdx4cv mice 27 ^,^ 31 ^-^ 33. The mdx/FVB mice showed the similar trend. Based on the phenotypic similarity between mdx/FVB mice and the original mdx mice, we conclude that mdx/FVB may serve as a good control for studying FVB-background transgenic mdx mice.

**Table 2. Contractile properties of FVB versus BL10 limb muscles:** a, values are from the Duan lab studies b, values are from 4-m-old BL6 mice

	FVB (3 to 6-m-old)	BL10 (2 to 8-m-old)	References
**Extensor digitorum longus**			
Specific twitch force (mN/mm^2^)	34.8±1.7 to 40.8±1.5	33.2±1.8^a^ to 46.5±3.7^a^	26, 27, 28
Specific tetanic force (mN/mm^2^)	189.0±8.7 to 202.0±3.4	185.4±5.7^a^ to 245.0±1.4^a^	26, 27, 28
Percent force decrease following 10 cycles of eccentric contractions	26.9±2.0 to 28.1±1.5	26.8±0.25^a^ to 32.0±3.2^a^	26, 27
**Tibialis anterior**			
Specific twitch force (mN/mm^2^)	67.7±3.6 to 75.3±3.7	41.7±1.6^b^	31
Specific tetanic force (mN/mm^2^)	241.9±8.2 to 255.0±7.0	~225 to 268.5±4.0^b^	12, 29, 30, 31
Percent force decrease following 10 cycles of eccentric contractions	39.0±1.9 to 44.2±1.9	23.8±3.6^b^	31

**Table 3. Contractile properties of mdx limb muscles on the FVB (mdx/FVB) versus BL10 background (mdx):** a, values are from the Duan lab studies b, Data are from 4-m-old mdx4cv mice

	mdx/FVB (3 to 6-m-old)	mdx (2 to 8-m-old)	References
**Extensor digitorum longus**			
Specific twitch force (mN/mm^2^)	24.5±2.3 to 27.3±1.2	26.6±1.2^a^ to 29.0±1.5^a^	26, 27, 28
Specific tetanic force (mN/mm^2^)	120.4±9.3 to 142.2±7.4	129.6±10.5^a^ to 138.5±5.6^a^	26, 27, 28
Percent force decrease following 10 cycles of eccentric contractions	47.5±2.5 to 55.5±1.8	46.5±4.5^a^ to 70.6±2.7^a^	26, 27
**Tibialis anterior**			
Specific twitch force (mN/mm^2^)	63.5±4.7 to 72.2±2.3	27.7±3.0^b^	31
Specific tetanic force (mN/mm^2^)	192.5±10.3 to 206.8±7.1	129.5±10.5^b^ to ~200	12, 29, 30, 31
Percent force decrease following 10 cycles of eccentric contractions	65.1±5.4 to 78.6±2.0	66.4±5.2^b^	31

Over the last two decades, mdx mice have been backcrossed to the background of at least six different inbred strains including albino, BALB/C, C3H, BL6, DBA/2 and FVB 34 ^-^ 40. Except for the DBA/2 background mdx mice 37, the dystrophic phenotype is rarely characterized in other backgrounds. It has become apparent that genetic background can significantly modulate the phenotype of single gene mutation in mice (Reviewed in 41 ^-^ 44). This feature has been used in genome-wide genetic analysis to identify the genetic modifiers that may account for the phenotypic differences in muscular dystrophy (reviewed in 45 ^,^ 46). The mdx/FVB strain described here may add in the research in this direction.

## Corresponding Authour

Dongsheng Duan Ph.D.

Department of Molecular Microbiology and Immunology

One Hospital Drive

Columbia, MO 65212

Phone: 573-884-9584

Fax: 573-882-4287

Email: duand@missouri.edu

## Competing Interests

The authors have declared that no competing interests exist.
